# Protein Diffusion in Mammalian Cell Cytoplasm

**DOI:** 10.1371/journal.pone.0022962

**Published:** 2011-08-19

**Authors:** Thomas Kühn, Teemu O. Ihalainen, Jari Hyväluoma, Nicolas Dross, Sami F. Willman, Jörg Langowski, Maija Vihinen-Ranta, Jussi Timonen

**Affiliations:** 1 NanoScience Center, Department of Physics, University of Jyväskylä, Jyväskylä, Finland; 2 NanoScience Center, Department of Biology, University of Jyväskylä, Jyväskylä, Finland; 3 MTT Agrifood Research Finland, Jokioinen, Finland; 4 Division Biophysics of Macromolecules, German Cancer Research Center (DKFZ), Heidelberg, Germany; Ohio State University, United States of America

## Abstract

We introduce a new method for mesoscopic modeling of protein diffusion in an entire cell. This method is based on the construction of a three-dimensional digital model cell from confocal microscopy data. The model cell is segmented into the cytoplasm, nucleus, plasma membrane, and nuclear envelope, in which environment protein motion is modeled by fully numerical mesoscopic methods. Finer cellular structures that cannot be resolved with the imaging technique, which significantly affect protein motion, are accounted for in this method by assigning an effective, position-dependent porosity to the cell. This porosity can also be determined by confocal microscopy using the equilibrium distribution of a non-binding fluorescent protein. Distinction can now be made within this method between diffusion in the liquid phase of the cell (cytosol/nucleosol) and the cytoplasm/nucleoplasm. Here we applied the method to analyze fluorescence recovery after photobleach (FRAP) experiments in which the diffusion coefficient of a freely-diffusing model protein was determined for two different cell lines, and to explain the clear difference typically observed between conventional FRAP results and those of fluorescence correlation spectroscopy (FCS). A large difference was found in the FRAP experiments between diffusion in the cytoplasm/nucleoplasm and in the cytosol/nucleosol, for all of which the diffusion coefficients were determined. The cytosol results were found to be in very good agreement with those by FCS.

## Introduction

Living cells are multifunctional organisms that exhibit remarkable dynamic phenomena including, e.g., cell motility, and vesicular, cytoplasmic and nuclear transport. The cytoplasm consists of a viscous liquid phase (the cytosol) and a non-liquid phase that will be called here the solid phase. The protein concentration in the cytoplasm has been estimated to be 100 mg/ml [1], and its total macromolecular concentration (proteins, lipids, nucleic acids, and sugars) can be as high as 400 mg/ml [2]. The cytoplasm can thus be described as a ‘molecularly crowded’ environment, where macromolecules can occupy 20–30% of its volume [3]. Its solid phase is composed of a dense network of cytoskeletal filaments and membrane structures such as, e.g., the endoplasmic reticulum (ER), Golgi apparatus, and mitochondria [4], [5]. Macromolecular diffusion in the cytoplasm can be severely restricted in such an environment [6]. The same applies to the nucleus [7]–[9] that is also composed of a liquid phase, the nucleosol, and a solid phase comprising, e.g., chromatin and proteinaceous nuclear bodies.

Diffusive motion of macromolecules and their binding-dissociation reactions with cellular organelles is a crucial component of cell function, which still need to be clarified. Laser scanning confocal microscopy (LSCM) has become very popular as it allows three-dimensional observation in living cells. LSCM can also be used to perform photo-manipulation experiments such as quantitative fluorescence recovery after photobleaching (FRAP). In FRAP, a region of the cell is exposed to high-intensity laser light, causing the fluorophores within that region to irreversibly lose their ability to fluoresce. Recovery of fluorescence in that region yields information about molecular diffusion and binding in the cell [10], [11].

Since the invention of FRAP, several analytical models have been developed to quantify the recovery of fluorescence and thereby diffusion and binding dynamics [12]–[17]. As the internal structure and conditions of the cell are difficult to include in such modeling, several assumptions are made of the system. These assumptions often include infinite, homogeneous fluorophore pools, fast bleaching compared to the time scales of the involved transport processes, and specific shapes of the bleach profiles, conditions that may be difficult to fulfill in FRAP experiments. Models have been suggested that account for diffusion during the bleach phase [15], [17], allow for arbitrary bleach profiles [16], [18], [19], or inhomogeneous distribution of fluorophores inside the cell [20] or of binding sites in the nucleus [21]. Recently the structure of ER [22] has been included when studying protein diffusion in the ER lumen. In these models, however, the constraints imposed by cellular structures have only been partly included.

Fluorescence correlation spectroscopy (FCS) is a method that probes the diffusion coefficient locally, while FRAP probes quite widely the cytoplasm (nucleus). Fluorescence fluctuation microscopy (FFM) combines FCS with LSCM, thereby being able to image the cell environment in which the FCS measurement is conducted. It is expected that the diffusion coefficients measured by FFM and conventional FRAP are quite different unless the internal structure of the cell is included in the latter. There are indeed large differences between the diffusion coefficients reported by FRAP and FFM measurements [14], [23], [24]. It is evident that, to improve our understanding of dynamic cell functions, better description of macromolecular motion in the cell, and thereby interpretation of experimental results, is called for.

We introduce therefore a completely new approach to protein dynamics in the cell based on describing their diffusive motion in a realistic three-dimensional representation of the cell generated from LSCM data. As it is neither useful nor possible to simulate the dynamics of all proteins of a species in an entire cell at a molecular level of detail, we rely here on mesoscopic methods to model protein distributions instead. This approach is applied here to analysis of FRAP data on the same cell. The model cell takes into account the internal structure of the cell using the inhomogeneous fluorophore distribution at equilibrium. We introduce the new methods by determining the diffusion coefficient of a freely-diffusing model protein, enhanced yellow fluorescent protein (EYFP), in two continuous cell lines, fibroblast-like Norden Laboratory Feline Kidney (NLFK) [25] and cervical carcinoma HeLa [26]. We also determine this diffusion coefficient by FFM for the same cell lines so as to be able to compare the results of the new FRAP analysis with those of FFM analysis. Recent studies using single particle tracking and fluctuation methods have shown that proteins may also undergo anomalous diffusion in the cytoplasm [27], [28] as well as in the nucleus [29]. These processes are, however, not considered here as the assumption that all diffusive processes are of Brownian nature suffices to interpret the measured (collective) FRAP data (for theoretical studies of anomalous diffusion see, e.g., [30], [31]).

## Methods

### Cell culture

Norden laboratory feline kidney (NLFK) [25] and HeLa [26] cells were grown in Dulbecco's modified Eagle medium (DMEM) supplemented with 10% fetal bovine serum (Gibco, Paisley, UK) at 37°C in the presence of 5% CO2. HeLa cells used for FFM measurements were grown as described in [23]. For live cell microscopy studies, the cells were seeded in 5 cm glass-bottom culture dishes (1.5 thickness, MatTek Cultureware, Ashland, MA). For FFM imaging and measurements, cells were transferred and transfected on 32 mm cover slips as described in [23]. The pEYFP-N3 construct was purchased from Clontech Laboratories Inc. (Mountain View, CA). Transfections were performed with the TransIT-LT1 reagent (Thermo Fisher Scientific Inc, Waltham, MA) according to the manufacturer's protocol. The intracellular localization of the nucleus was visualized by chromatin binding fluorescent histone H2B-ECFP. Cells were transfected with a H2B-ECFP expression vector 24 h after cell seeding.

### FRAP experiments

**Figure 1:**
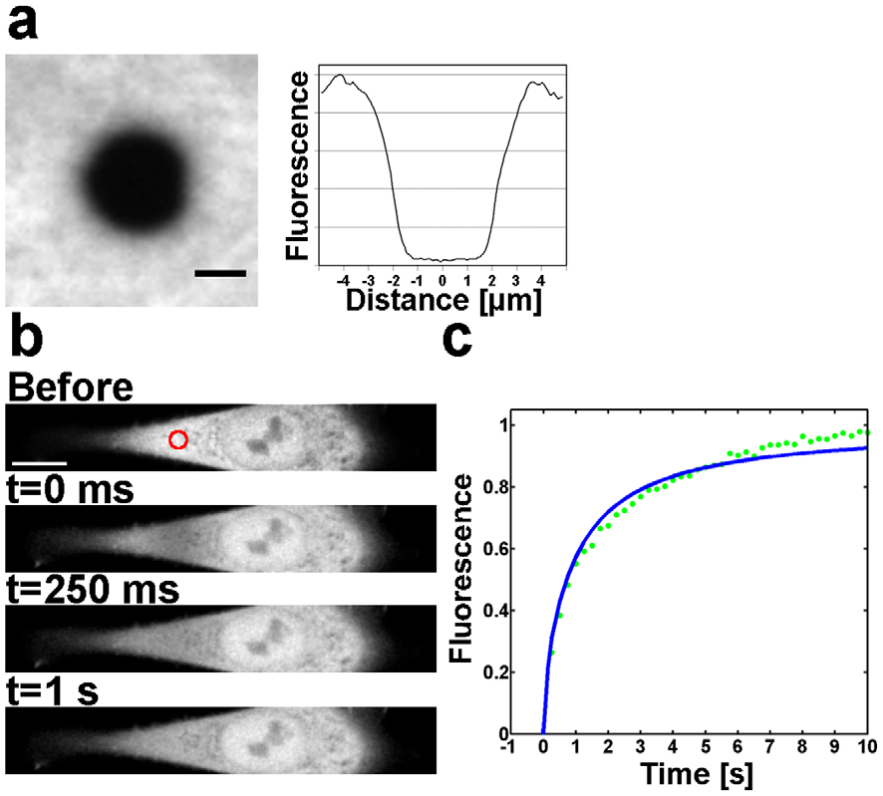
FRAP experiment in an NLFK cell stably expressing EYFP. (a) The average (n = 10) bleach profile measured on fixed cells expressing EYFP. Scale bar 2 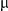
m. (b) Fluorescence distribution before the bleach pulse and the position of the circular bleach area (diameter 20 pixels, FWHM 3.7 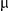
m). Subsequent images show the fluorescence distribution immediately (t = 0 ms), and 250 ms and 1 s after the bleach pulse. Scale bar 10 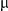
m. (c) The measured recovery curve (Axelrod normalization) and a fit by the free-diffusion model of Soumpasis.

The FRAP experiments were performed on a laser scanning confocal microscope FV1000 with an IX-81 microscope frame (Olympus, Tokyo, Japan) using an Olympus UPLSAPO 60× (NA = 1.2) water immersion objective. The sample stage was heated to 37°C prior the experiments. To image the cell geometry, a confocal stack was acquired before and after the FRAP experiment. The voxel size was adjusted to (200 nm)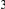 or (150 nm)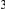. The pinhole size was adjusted to 1 Airy unit. The 514 nm laser line was used for EYFP excitation and the emitted fluorescence was detected using a 530–600 nm band pass filter. Imaging was performed with a laser intensity of 0.1–2 For bleaching a circular (r = 1.85 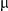
m and 2.83 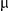
m) region of interest (ROI) was defined in the middle of the cytoplasm. As bleaching times in FRAP are usually rather large compared to the time scales of the measured diffusion processes, the region of the cell, which is actually bleached, is usually larger than the defined ROI. The size of the actually bleached region and its intensity distribution were measured by bleaching fixed cells (Fig. 1). ImageJ [32] was then used to construct an average shape and intensity profile of that region.

**Figure 2:**
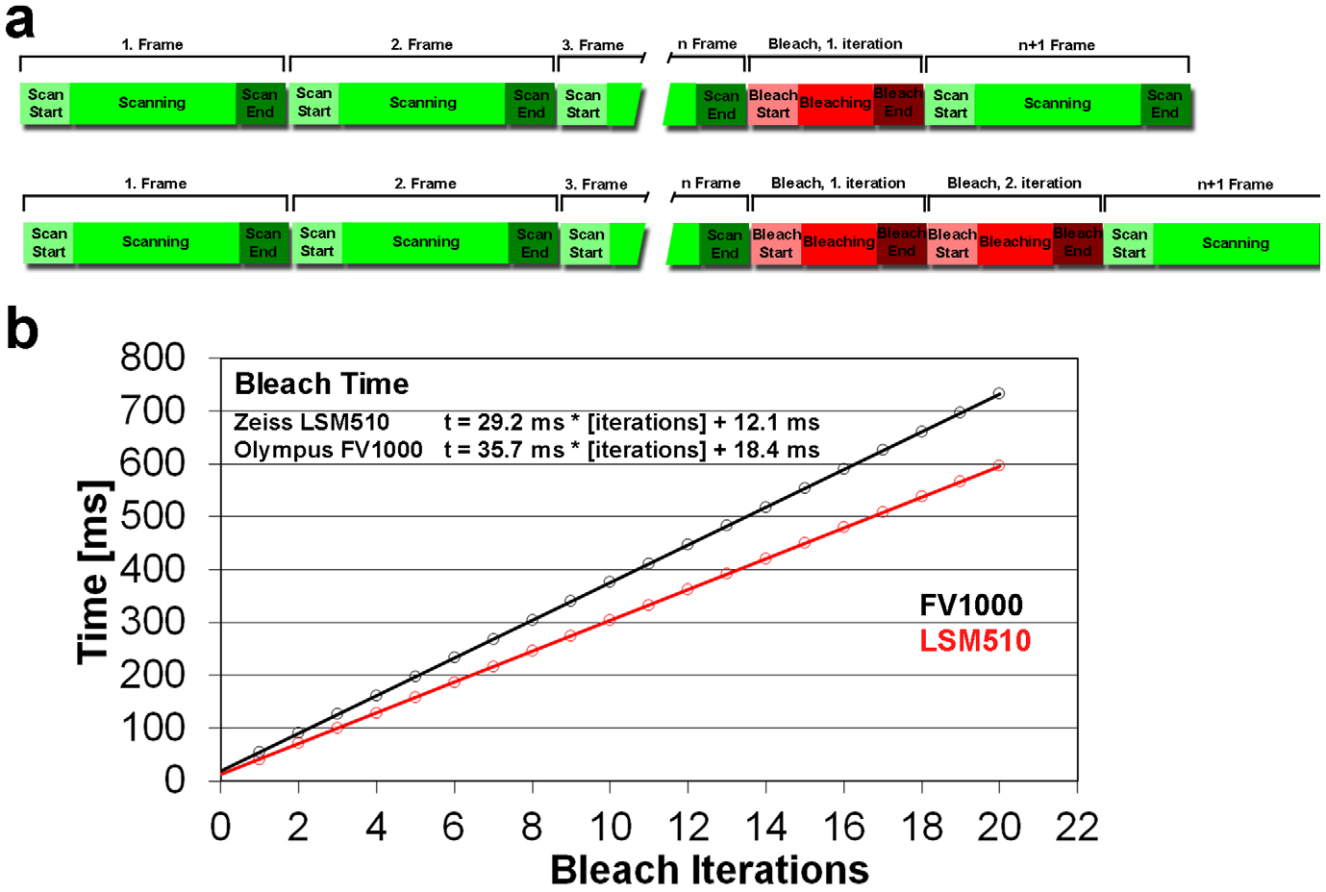
Duration of the bleaching phase in FRAP experiments for two confocal microscope setups. (a) Schematic representation of the confocal imaging combined with bleaching phase. (b) Bleach phase duration as a function of the number of bleaching iterations for the two confocal microscope setups used in the study, red is the results for a Zeiss LSM510 and blue for an Olympus FV1000 confocal microscope.

The duration of the bleach process was measured by performing FRAP experiments in which 10 images were collected before the bleach pulse and 1 after the pulse. The bleach time was extracted by measuring the time when the frames immediately before and after the bleach pulse were taken. The average imaging time of one frame was subtracted, and the duration of the bleach process was plotted as a function of iterations (bleaching time), yielding a linear slope. In the LSCM used (Olympus FV 1000), the shortest possible bleach procedure (1 iteration) lasted 36 ms with an additional relay of 18 ms before the next image scan, amounting to 54 ms for the entire process. To achieve enough bleaching for the data analysis, 10 iterations were performed (Fig. 2), and the laser intensity was set to 100% by using an acousto-optical tunable filter.

### Image processing

The raw images of the confocal microscope were converted to 8-bit grey scale images. Only linear adjustments of the image brightness and contrast were performed, avoiding saturation. The gray-scale images were colored with an appropriate look-up table and converted to RGB images.

### Conventional FRAP analysis

The fluorescence recovery was analyzed in the circular regions described above using the ImageJ [32] and Excel (Microsoft, Redmond, USA) software. Before the measurements, the FRAP data were convoluted with a 3×3 Gaussian kernel. The data were exported to Excel where their normalization was performed. For normalization two different methods were used. The first normalization (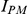) used was that of Phair & Misteli [10]:
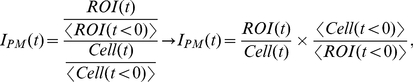
(1)where 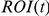 is the local fluorescence intensity in the bleached region at time 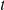, 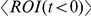 is the time average of the local fluorescence intensity of the whole bleached region before the bleach pulse, and 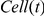 and 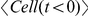 are the respective quantities for the entire cell. The second normalization (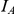) used was that by Axelrod et al. [12]:
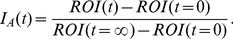
(2)Here 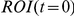 is the local fluorescence intensity of the bleached region immediately after the bleach phase and 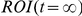 is the fluorescence intensity after recovery. The time of full recovery can be difficult to determine and requires long imaging times. If the fluorescence is not completely recovered at the end of the experiment, this normalization will lead to too rapid recovery. Therefore we modified the Axelrod normalization (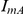) such that it could also be used for partial recovery:
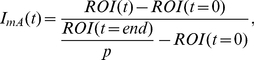
(3)with 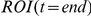 the fluorescence intensity at the end of the experiment and 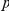 the recovery ratio at that time. By definition, the Phair & Misteli normalization always converges to one at full recovery. At partial recovery the value of the Phair & Misteli normalized data can thus be used as the value 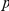 needed in the modified Axelrod normalization.

The fluorescence intensity data were fitted by the free diffusion model of Soumpasis [13], [14]:
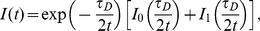
(4)where 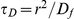, 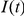 is the normalized fluorescence, 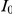 and 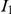 are modified Bessel functions, 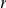 is the radius of the bleached region, and 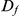 the diffusion coefficient of the fluorescent species [33].

### FFM measurements

The Fluorescence Fluctuation Microscope (FFM) measurements were conducted with a self made setup in Heidelberg [34]. FFM is a combination of Fluorescence Correlation Spectroscopy (FCS) and Laser Scanning Confocal Microscopy (LSCM). It has an FCS module with a galvanometer scanning unit, attached to the side port of an inverted Olympus IX-70 microscope (Olympus, Hamburg, Germany) equipped with an UplanApo/IR 60× water immersion objective, with a numerical aperture (NA) of 1.2 [34], [35].

EYFP was excited with the 488 nm line of an argon-krypton laser from CVI Melles Griot (Bensheim, Germany). The emitted fluorescence from EYFP was recorded between 515 and 545 nm with an avalanche photodiode (APD) (SPCM-AQR-13, PerkinElmer, Wellesley, USA), after passing through appropriate dichroic mirrors and filters for spectral separation and selection. FCS measurements were carried out at laser intensities of 5 to 9 kW cm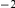, and the laser power was adjusted using a polychromatic acousto-optical modulator AOTF Nc (AA Opto Electronic, France). The signals from APD were fed into an ALV-5000/E correlator card (ALV Laser GmbH, Langen, Germany) which recorded the intensity fluctuations and calculated their associated autocorrelation function almost in real time.

The system was carefully calibrated as described in [23] to allow for precise and reproducible measurements.

### Construction of the digital model cell and FRAP recovery simulations

For each FRAP experiment we obtained two sets of data: a 3D stack of images depicting the intensity profile of EYFP and H2B-ECFP (histone H2B linked to enhanced cyan fluorescent protein)in the cell before the bleach, and a stack of 2D images depicting a certain cross-section of the cell during the FRAP measurement, 10 frames before the bleach and the rest of the frames from the fluorescence recovery phase. After de-noising the 3D stacks, we used the threshold function of the ImageJ program to segment in the cell the cytoplasm and nucleus using the EYFP and H2B-ECFP stacks, respectively. The nuclear envelope was then generated as a two pixel wide layer between the cytoplasm and nucleus using a self-made code.

The spatial resolution of LSCM, about 200 nm, did not allow segmentation of the more detailed structure. As this ‘fine structure’ obstructs protein motion and thus affects protein diffusion, we considered the cytoplasm (nucleoplasm) as an ‘effective porous medium’ that is immobile during a FRAP measurement.

**Figure 3:**
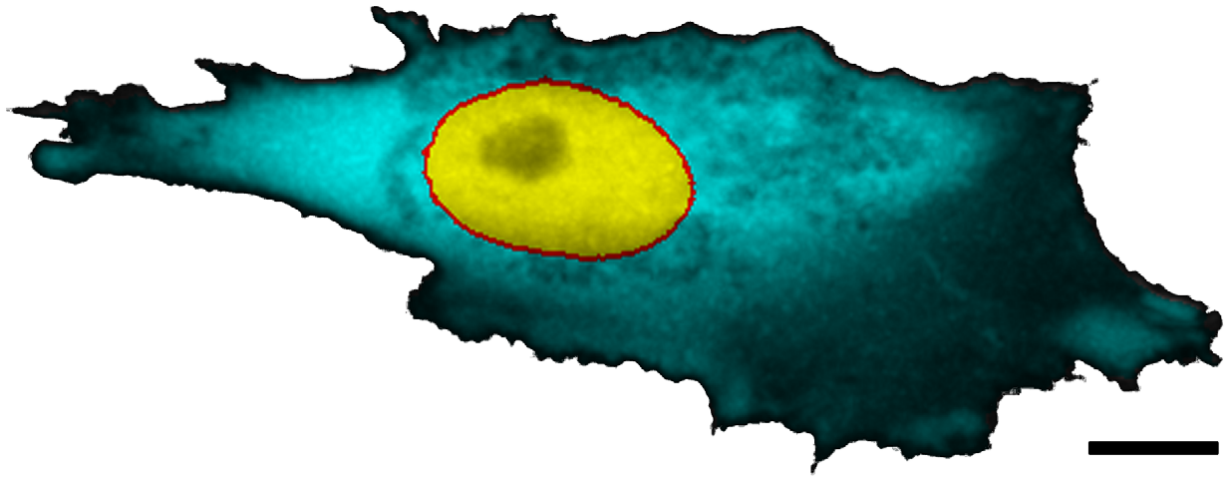
2D cross-section of a digital model cell. The different regions of the cell are displayed in different colors (cytoplasm in cyan, nucleus in yellow, and nuclear envelope in red). The color intensity at each pixel refers to the effective porosity (volume fraction available for protein motion) at that point in the cell. Scale bar 10 µm.

From diffusion in porous media [36] (see Discussion for a simple example), we know that one must distinguish diffusion in the liquid phase (cytosol/nucleosol with 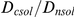) from that in the medium with constrained motion (cytoplasm/nucleoplasm with 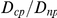 such that, approximately, 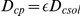 and 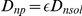, where 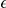 is the porosity of the medium. As 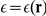, 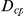 (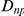) is a spatially varying ‘effective’ diffusion coefficient. A separate diffusion coefficient (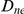) was assigned to the nuclear envelope described as a permeable membrane. The porosity of the medium was made visible by the heterogeneous equilibrium distribution of fluorophores (proteins), low fluorescence intensity meaning high concentration of ‘solids contents’, and was thus deduced from the equilibrium fluorescence intensity 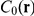 (the 3D EYFP stack) such that 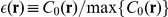. A 2D cross-section of a typical digital model cell is depicted in Fig. 3.

Due to the low imaging speed of the LSCM used, only a 2D cross section of the cell was imaged during the FRAP experiments. In order to be able to simulate the FRAP recovery in the entire cell, the initial bleach profile had to be extrapolated vertically into the rest of the digital cell. To this end we first determined the relative fluorescence reduction 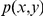 by dividing pixel-by-pixel the first post-bleach image with an average (for noise reduction) of all 10 pre-bleach images of the experimental FRAP data. To enforce the theoretical range of 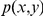 between zero and one, greater valued pixels owing to the noisiness of the experimental data where set to one (flat field correction). The 3D bleach profile was then obtained by multiplying each cross section of 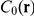 with 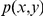.

The cross section of the cell, which was imaged during the FRAP experiment, was determined by cross-correlating each frame of 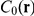 with an average of the 10 pre-bleach images of the FRAP stack. The cross-correlation coefficient showed a clear maximum that identified the right cross section.

### The lattice-Boltzmann method

The spatial and temporal evolution of diffusion processes is described by the diffusion equation,
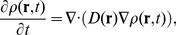
(5)where 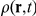 is the concentration of diffusing particles and 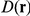 their possibly locally varying diffusion coefficient. Note that Eq. 5 only accounts for Brownian diffusion processes, in the case of anomalous diffusion, a fractional version of this equation may be used [30]. In Eq. 5 the term
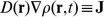
(6)is the local diffusive flux of particles. In the present realization we introduce the impermeable solid component in the cytoplasm and nucleoplasm such that an additional flux term, 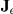, is added to the flux. This term takes care of removal of particles from the non-accessible regions. The construction of this flux is discussed below. The total flux of diffusing particles is now given by 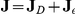, and Eq. 5 can be expressed in the form
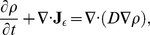
(7)which is an advection-diffusion equation, where the (local) advective component is given by the additional flux.

In the case of complicated boundary conditions it is, for a numerical realization of Eq. 5, more convenient to start at a somewhat more microscopic level. We thus consider instead the Boltzmann equation [37]. Suitably chosen discrete versions of the Boltzmann equation, in which space, time and velocity are all discrete [37], allow very effective numerical implementations. In the single relaxation time (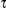) approximation a discrete Boltzmann equation for the distribution function 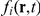 of particles at point 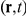 moving with velocity 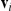 in the (lattice) direction 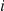, called the lattice-Boltzmann (LB) equation, is given by
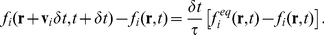
(8)Here the left-hand side describes the streaming of particles during a time step 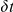, and the right-hand side models the relaxation of their distribution function towards its local equilibrium, 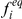, on a time scale set by the relaxation time. We have now a three dimensional space and choose a simple cubic lattice with nearest neighbor links only (particles can only move to these nearest neighbors during one time step, which is enough in the case of the diffusion equation [38]). We also allow the particles not to move, and have therefore seven possible velocities (the so-called D3Q7 model [37]) for the particles: 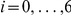. In this case of an advection-diffusion equation the equilibrium distribution function is given by
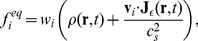
(9)in which 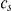 is a free numerical parameter (in units of velocity) that determines the proportion of the rest particles, 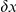 is the lattice spacing and 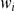
's are the D3Q7 weight factors for different discrete velocities: 
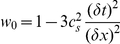
 for the rest particles and 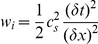 for the other discrete velocities. The second term in Eq. 9 accounts for removing of particles away from the non-accessible regions. The concentration of particles is given by 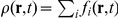, and it satisfies (in the continuum limit) Eq. 7 when [38] the diffusion coefficient is given by
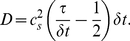
(10)This diffusion coefficient can be tuned either by changing the relaxation time 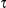, parameter 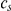, or time step 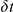. For numerical convenience we fix parameters 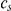 (such that 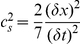) and the relaxation time 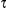, and change the diffusion coefficient by tuning the time step.

When applied to modeling a FRAP experiment, the particle density 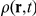 is interpreted as the fluorophore concentration (fluorescence intensity) 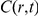. The additional flux will cancel the diffusive flux into the non-accessible regions filled by membranes, which arises from the concentration gradients in the fluorophore distribution, such that the observed non-homogeneous fluorophore distribution before the bleach will also stay at equilibrium in the simulated model cell. The equilibrium distribution is obtained by setting, at all points 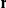 in the cell,
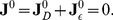
(11)Hence the additional flux at equilibrium must be of the form
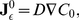
(12)where 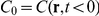, i.e., it is the (equilibrium) fluorophore distribution before the bleach. During a (FRAP) simulation, the magnitude of this flux at any point in the cell will depend on the actual concentration at that point, which varies in time. Thus, at a given time 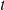, it can be expressed in the form
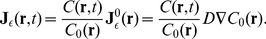
(13)


A numerical code was constructed along the lines indicated above, which was capable of simulating the spatial and temporal evolution of the fluorescence intensity in the digital realization of the cell actually measured in the FRAP experiments. When the distribution of fluorescence intensity as measured right after the bleach was taken as the initial condition in the simulation, such simulations could very accurately reproduce the experimentally observed fluorescence recovery.

### FRAP data analysis

At every time step during FRAP recovery, the fluorophore distribution was simulated in the whole model cell, and it was recorded in the same cross section as in the measurement. We compared the experimental and simulated frames by cross correlation such that the cross-correlation coefficient was given by
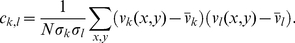
(14)Here the subscripts 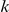 and 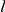 refer to the two series of frames to be compared, 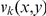 is the pixel intensity of frame 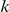, 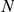 is the number of pixels, and 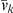 and 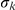 are the average intensity and standard deviation of image 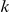, respectively.

The experimental and simulated frames could be compared directly using this algorithm. In practice however, the cross-correlation results were improved greatly if the cell background was removed from all the images (similar to the construction of 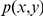 above), and a mask was used to restrict the analysis to the cell interior. By these manipulations we minimized the perturbing effects of cell motion and deformation.

**Figure 4:**
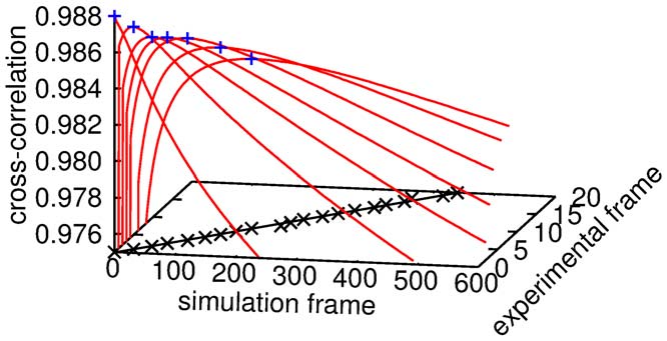
Visualization of the cross-correlation fitting of corresponding frames. For a given experimental image 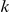, the cross-correlation coefficients 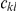 (red) each have a global maximum 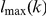 (blue crosses). By tuning the parameters 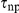 and 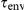, 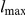 becomes a linear function of 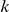 (black crosses and curve), whose slope determines the simulation time step 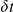. The deterioration of the maximum in 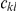 as a function of the experiment frame number stems from the broadening of the bleach profile, which inevitably decreases the relative difference between adjacent frames. Ultimately, this relative difference limits the amount of analyzable experimental frames, which may limit the applicability of the method to slow enough diffusion processes.

In the simulations we used three relaxation times to describe the different liquid phases of the cell, the cytosol (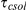), the nucleosol (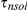), and the effective substance of the nuclear envelope (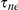). Of these three, we fixed 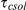 for numerical convenience, and the other two were then free parameters. The simulation time step was also a free fitting parameter that eventually determined, together with the values for the three relaxation times, the diffusion coefficients. For a given experimental frame 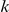, the 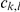 of Eq. 14 showed a global maximum as a function of the simulation frame number 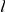, denoted by 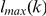. The real and digital cells were assumed to correspond to each other when 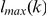 was a linear function of 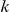. By varying the values of 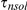 and 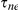, the linearity of 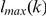 was maximized, and the slope of 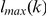 directly related the simulation time step 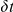 to the time step 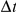 used in the experiment (See Fig. 4). These values were then used to determine the values for 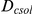, 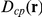, 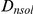, and 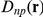. Finally, we also compared the measured and simulated fluorescence recovery curves for an additional check of consistency.

### Validation of methods

**Figure 5:**
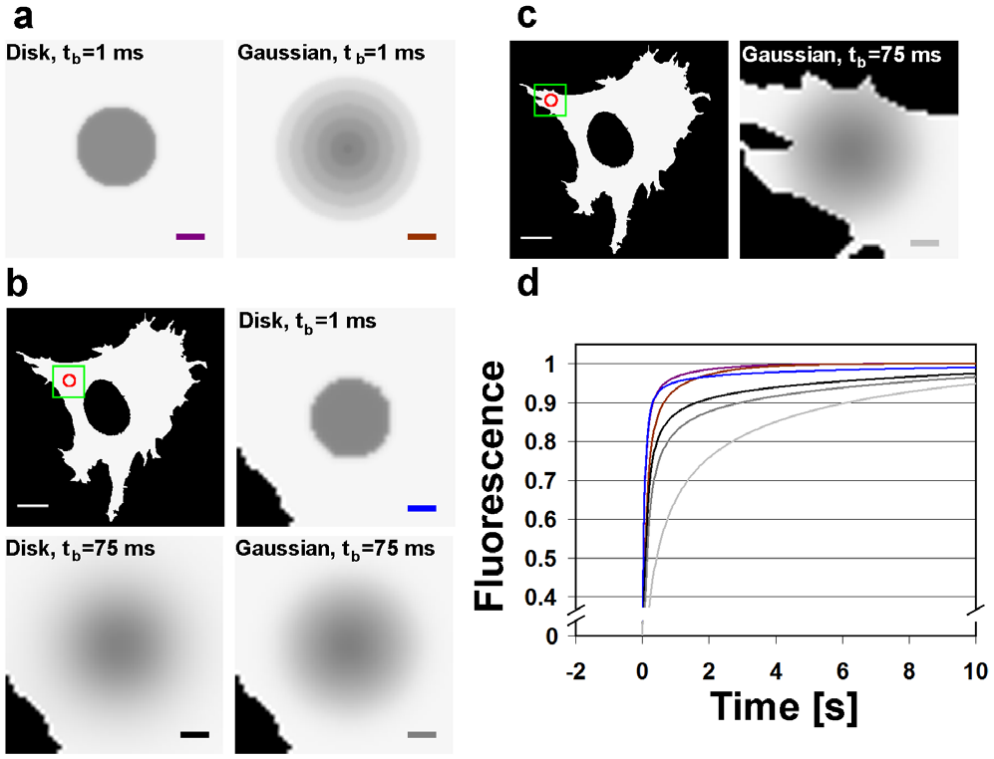
Simulated Virtual Cell data for FRAP experiments with a particle diffusion coefficient of D = 25 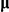
m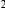/s. Simulations are for two different bleach locations, different bleach phase durations, and different bleach-laser profiles. (a) A bleached region in the middle of an isotropic environment immediately after a 1 ms bleach pulse with either a cylindrical (diameter 3.7 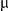
m) or Gaussian bleach-laser profile (FWHM 3.7 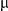
m). (b) A cross section of the cell with the bleached region far away from the cell boundaries and the nucleus. The blow-up images show the bleached region after 1 ms and 75 ms bleach pulses for the cylindrical bleach profile, and after a 75 ms bleach pulse for the Gaussian profile. (c) A bleached region near the cell boundary and immediately after a 75 ms bleach pulse for the Gaussian bleach profile. (d) The recovery curves for an isotropic environment and 1 ms bleach time with a cylindrical (purple) or Gaussian (dark green) bleach profile, for a real cell geometry with a cylindrical bleach profile and 1 ms (blue) or 75 ms (black) bleach time, with a Gaussian bleach profile and 75 ms (dark gray) bleach time, and for a bleached region near the cell boundary with a Gaussian bleach profile and 75 ms bleach time (light gray). Scale bars 10 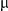
m.

In order to test the performance of conventional and the new data analysis methods introduced here, we used the Virtual Cell software [39] to produce data on quasi-2D FRAP experiments with known diffusion coefficients of 10, 25, 40, and 55 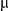
m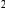/s. The bleaching process was modeled as a laser light induced reaction whose creation rate can be described as
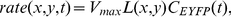
(15)where 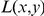 is the distribution of laser intensity in the simulation geometry, 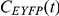 is the concentration of the molecules which are bleached, and 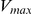 is the maximum reaction rate. The profile of the laser pulse was either cylindrical with a sharp boundary at a radius of 1.85 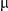
m, or a Gaussian with an HWHM of 1.85 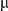
m (Fig. 5 a). The length of the bleach pulse in the fast and slow bleach simulations was adjusted to 1 ms and 75 ms, respectively. The time lag between the bleach and first recovery data point was 0 ms in the fast and 25 ms in the slow bleach simulations. The simulation time step was set to 0.1 ms or 1 ms, and the fluorescence intensity was recorded at 20 ms intervals. The recovery data were normalized as in the FRAP experiments.

We first produced data with different bleach profiles on a geometry (25 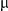
m×25 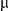
m, with a thickness of 1 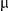
m and a pixel size of 100 nm) that very nearly conformed to the assumptions made in the Soumpasis method. Next we generated similar data on fluorescence recovery with different bleaching times in two different locations within a 2D digital cell outline determined from an LSCM image of an NLFK cell, with a thickness of 1 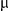
m and a pixel size of 200 nm (Fig. 5 b and c).

**Figure 6:**
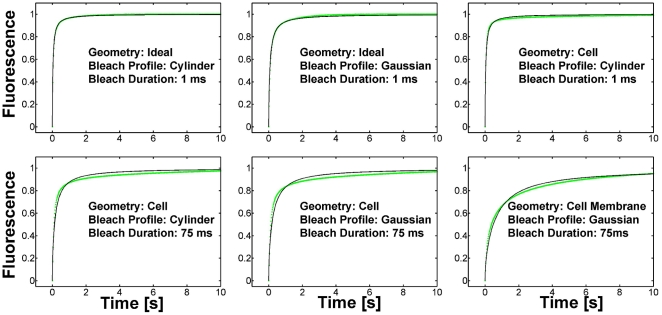
Virtual Cell simulation results for different FRAP experiments (blue) and their fits by the free diffusion model of Soumpasis (green). In the upper panels the bleach duration is very short, 1 ms, while the simulation geometry and bleach profile are varied. In the lower panels the bleach duration is much longer, 75 ms, and bleaching is done in a 2D outline derived from a real cell, either in the middle of the cell or near the plasma membrane.

As expected, in the ideal case the Soumpasis method recovered the correct value for the diffusion coefficient, especially at the low end of the values used. High diffusion coefficients produced some variation as the size of the periodic box had then a detectable effect on the results. The biggest difference was found for a Gaussian bleach profile placed near the boundary of the cell outline, and a bleach time of 75 ms. In this case the Soumpasis-method diffusion coefficient was 1.56 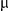
m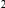/s, while the correct value was 25 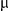
m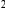/s, a difference by a factor of about 20 (see Fig. 6 for example recovery curves in different experimental conditions).

We then analyzed the same Virtual Cell data by the new LB method introduced here. We found very good agreement in all cases between the correct diffusion coefficient and the one obtained with the LB method, with a maximal deviation of 6%.

We also investigated the effect of data normalization on the FRAP results. In the Soumpasis method data are normalized as in Axelrod et al. [12]. However, the normalization introduced by Phair & Misteli [10], designed so as to include fluorescence loss in the imaging phase, is also very often used. In general, PM normalization increases the diffusion coefficient obtained. This increase seems, however, to be an artefact which arises from the fact that the recovery curve in the Soumpasis method begins at zero and asymptotically approaches one, but when the PM normalization is used, the initial intensity in the recovery phase can be anything between 0 and 1. This problem was demonstrated with the Virtual Cell data for which the Soumpasis method with Axelrod normalization gave the correct result. If we used instead the PM normalization, diffusion coefficients about four times too big were found. A full account of all the analyses done of the various Virtual Cell data is given in Table 1.

**Table 1:** Validation of methods: results.

Geometry	Homogeneous			Homogeneous			Cell		
Bleach profile	uniform			Gaussian			uniform		
Bleach duration	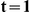 ms			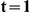 ms			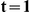 ms		
Used									
normalization	Phair		lattice-	Phair		lattice-	Phair		lattice-
method for	&	Axelrod	Boltz-	&	Axelrod	Boltz-	&	Axelrod	Boltz-
data analysis	Misteli		mann	Misteli		mann	Misteli		mann
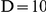	44.5	10.0	9.9	20.9	4.6	9.8	33.7	10.6	10.2
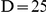	96.8	24.5	24.2	49.4	11.8	24.2	73.6	21.6	25.6
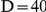	124.0	38.7	38.6	75.0	18.9	38.7	107.7	31.5	40.9
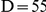	162.3	50.5	53.4	99.3	26.1	51.9	139.2	41.3	56.5

Comparison between the free-diffusion method of Soumpasis and the lattice-Boltzmann method using fluorescence recovery data produced under varying experimental conditions with the Virtual Cell software. Results are shown for the different conditions simulated, the different normalization methods used in the free-diffusion method, and for the lattice-Boltzmann method.

## Results

### FRAP analysis

We performed FRAP experiments on EYFP-expressing NLFK and HeLa cells. When the measured recovery data were analyzed by the Soumpasis method, we found a cytoplasm diffusion coefficient of 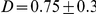 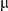
m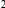/s (n = 8) for the NLFK cells and 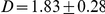 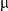
m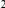/s (n = 13) for the HeLa cells.

**Figure 7:**
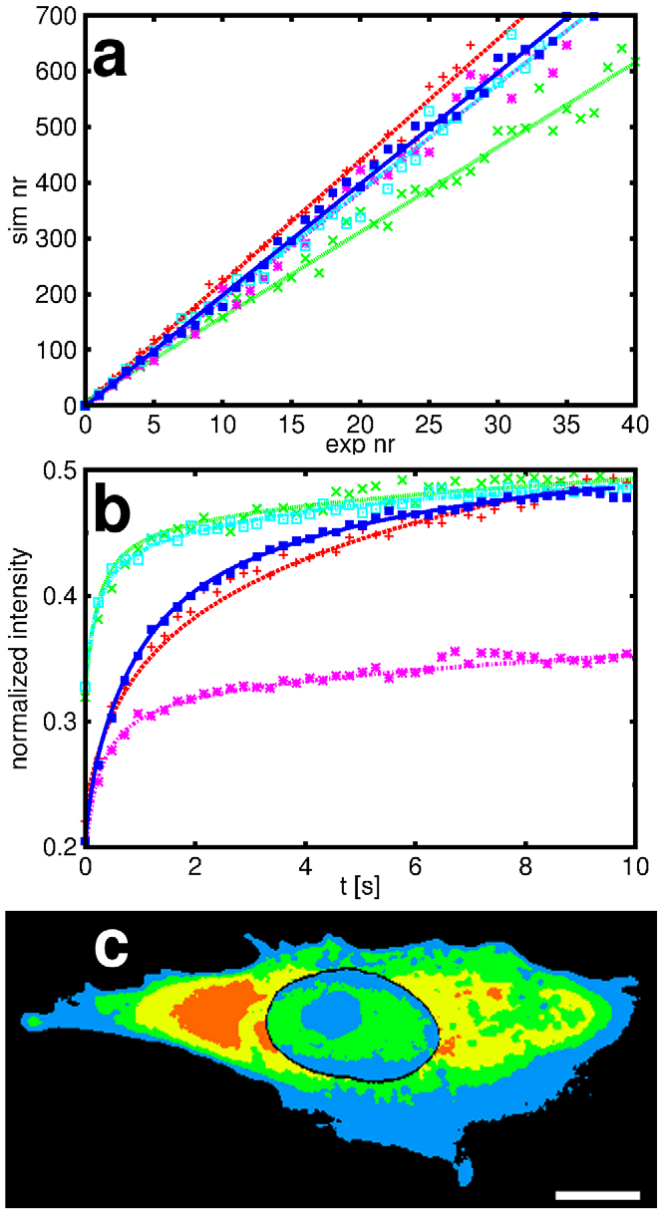
Results of FRAP analysis by the new method. (a) Correlation between experiment and simulation. Data points correspond to the function 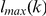 and the lines of the same color show the linear fit through the data. (b) Measured (data points) and simulated curves (continuous curves) of fluorescence recovery at the bleached ROI. The data were normalized by the maximum pixel value of the provided image data. Curves of the same color in (a) and (b) are taken from the same measurement. (c) Map of the local cytoplasm and nucleoplasm diffusion coefficients in a cross section of an NLFK cell. Scale bar 10 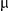
m.

In the new methods introduced, excellent correlations were found between experimental and simulated frames, and the corresponding fluorescence recoveries were also highly consistent (Fig. 7 a). The resulted *cytosol* diffusion coefficient, 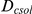, was 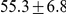 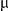
m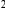/s for the NLFK (n = 12) cells and 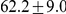 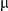
m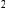/s for the HeLa (n = 13) cells. The difference in the liquid phase properties (‘viscosity’) of these cells is statistically significant (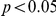), which indicates that they have different macromolecular concentrations. The *average cytoplasm* diffusion coefficients, 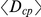, were 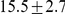 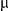
m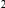/s for the NLFK and 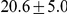 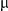
m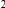/s for the HeLa cells, being thus quite similar. In both cell lines the cytoplasm diffusion coefficient, 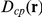, varied significantly (Fig. 7 c).

The emphasis was here on the cytoplasm, but the method automatically produced diffusion coefficients for the nucleus. The *nucleosol* diffusion coefficients, 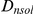, were found to be 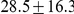 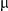
m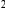/s for the NLFK and 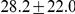 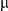
m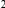/s for the HeLa cells, while the *average nucleoplasm* diffusion coefficients, 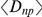, were 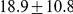 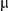
m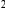/s (NLFK) and 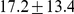 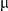
m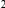/s (HeLa). In these values the uncertainty is obviously rather large as the measurements were not optimized here for their accurate determination. They can, however, be already used for qualitative conclusions.

### FFM analysis

**Figure 8:**
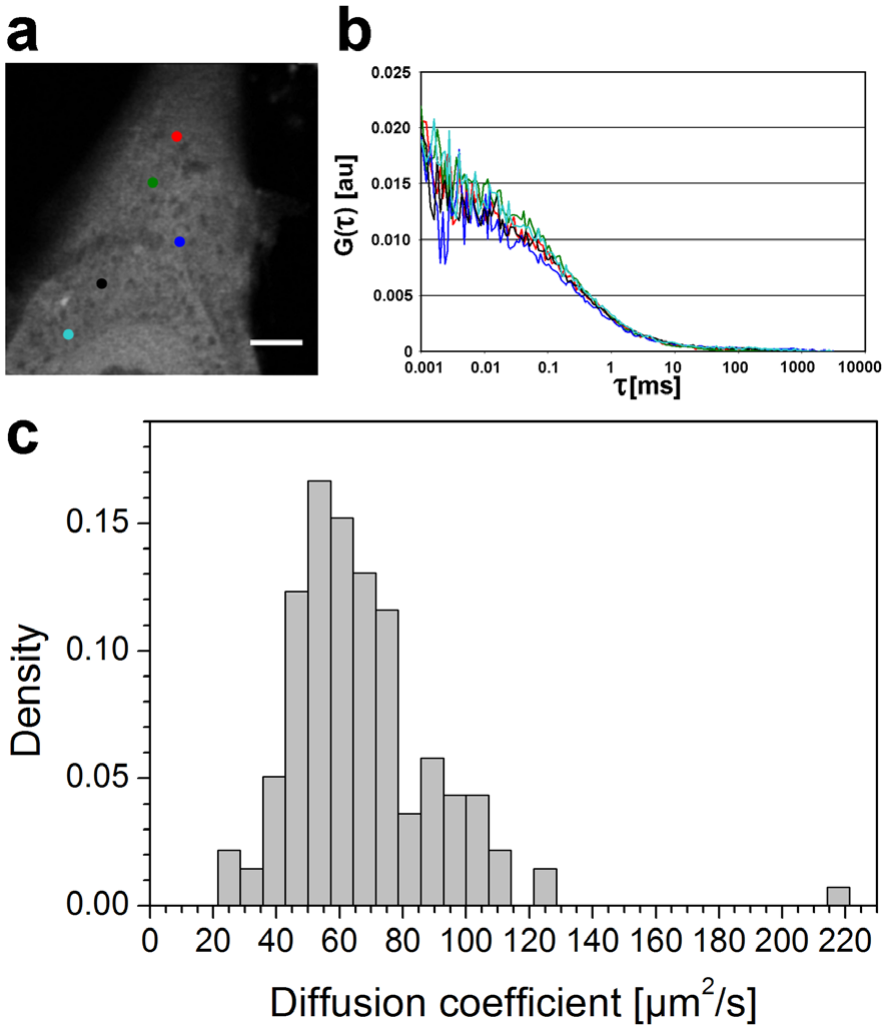
FFM results for NLFK cells. (a) An image of a cell taken before the fluorescence fluctuation measurements. The marked dots denote the points measured. Scale bar 5 m. (b) Autocorrelation curves of the measurements. The colors of the lines correspond to those of the measured point. (c) Distribution of the measured diffusion coefficients of the fast component (44 cells and 138 points).

As a local measurement technique for gaining further insight into the protein diffusion dynamics, we used FFM (Fig. 8). We measured the cytoplasm diffusion coefficient at 1 to 6 points inside 44 NLFK cells (138 measurement points in total) and at 1 to 9 points inside 50 HeLa cells (198 points in total), from the same cell lines as in the FRAP experiments. The data were fitted by a two diffusing components model [23], whose fast component was estimated to correspond to the cytosol diffusion coefficient determined by the new method introduced here (see the discussion below). In this way we obtained for the cytoplasm an average diffusion coefficient of 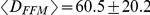 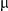
m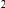/s for the NLFK and 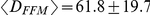 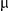
m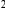/s for the HeLa cells. Note that these values are indeed very similar to the ones found by the new FRAP analysis method for the cytosol, 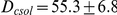 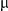
m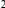/s for the NLFK and 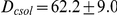 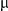
m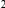/s for the HeLa cells.

## Discussion

We introduced a new method for modeling protein motion, i.e., evolution of fluorescence intensity in the case of fluorescent proteins, in the entire cell. This method was based on first constructing a three-dimensional digital representation of the cell, which included the cytoplasm, nucleoplasm and nuclear envelope. We furthermore included the effect of internal structures that obstruct protein motion by describing the two cellular compartments as porous media. The equilibrium fluorescence distribution was used to identify the degree by which protein motion is locally obstructed.

The diffusive motion of proteins could then be numerically simulated in a realistic cellular environment. Here we used the lattice-Boltzmann (LB) method for the numerical realization of the diffusion equation. Other methods could also have been used, but the LB method allows an easy implementation of the boundary conditions and an easy generalization to binding-dissociation processes that we intend to include later.

The new modeling instrument was applied to model FRAP experiments that are known to produce typically much lower diffusion coefficients for proteins, when conventional modeling is used to interpret the measured data, than FCS experiments. The fluorescence intensity distribution measured right after the bleach process was used as the starting point for the simulated (post-bleach) evolution of that distribution in the entire cell. Thus, in the new method, problems related within conventional modeling to, e.g., intensity normalization, finite volume of fluorophore distribution, and internal membrane structures, were all removed.

As it was expected that the internal membrane structures in the cytoplasm (and nucleoplasm) play an important role in protein transport in the cell, special emphasis was put on properly describing their effect. As described above, such structures could be included as non-accessible regions for protein motion by describing both the cytoplasm and the nucleolasm as porous media. This is not, however, enough. The heterogeneous fluorescence intensity in the cytoplasm/nucleoplasm was interpreted such that it was homogeneous in their liquid phases in which the diffusive motion of proteins only takes place. Distinction was therefore made between diffusive motion in the liquid phases and in the whole cytoplasm/nucleoplasm. The former diffusion coefficients are intrinsic properties of the liquid phases independent of where bleaching is performed. In contrast with this, the cytoplasm/nucleoplasm diffusion coefficient depends on the local membrane structures in and near the region of interest, and varies appreciably.

**Figure 9:**
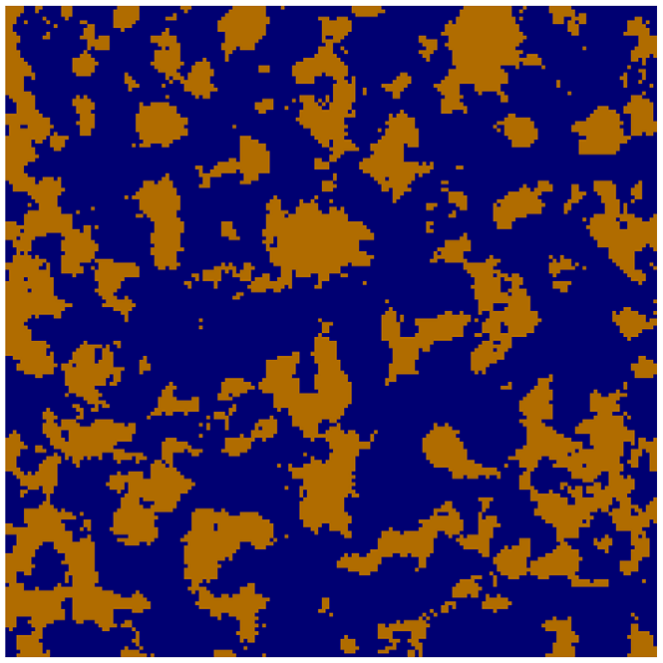
A cross section of an artificial porous medium that is homogeneous in the third dimension. Its porosity is 68%. The liquid phase is marked blue and the impermeable solid phase is brown.

In order to better understand the distinction between the two types of diffusion coefficient, consider an artificial porous medium a cross section of which is shown in Fig. 9. It can be interpreted as a small region of the cytosol as seen in a confocal microscope image of a cell. The liquid phase is marked blue and the impermeable solid phase is dark brown (its morphology does not represent that of membrane structures in the cytoplasm). We consider diffusion of tracer molecules across the shown structure (homogeneous in the third direction) such that their diffusion coefficient in the liquid phase is set to be 50 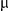
m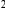/s. Diffusion from left to right across the shown medium results in an effective diffusion coefficient of 29 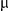
m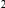/s. This is just a bit smaller that porosity (68%) times the diffusion coefficient in the liquid phase (50 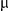
m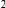/s) because of tortuosity effects (migration paths are in practice longer than the thickness of the region). Inclusion of tortuosity effects is, however, difficult in cellular transport, and they are expected to be rather small on the average.

Diffusion of a small non-binding (non-specific binding was assumed to be negligible) fluorescent protein was then analyzed by FRAP, using the new as well as conventional methods, and by FFM, and two different cells were used in these analyzes for generality.

Using FRAP combined with the new analysis method, the cytosol diffusion coefficients were found to be different in the NLFK and HeLa cells, indicating a different macromolecular concentration (‘viscosity’) in the cytoplasm. In both cases the *average cytoplasm* diffusion coefficient was about a third of that of *cytosol*, indicating a similar relative amount of membrane structures in the cytoplasm. FFM analysis of the cytoplasm resulted in diffusion coefficients that were very similar to those found by FRAP for the cytosol. This method probes rather closely the properties of the liquid phase, but if there are ‘solid phase’ structures near the region analyzed, they however affect [7], [8] the result of the measurement. This phenomenon is evidenced by the sizable local variations in the FFM results (in the cytoplasm and in the nucleus [23]). They prevented the detection here of the difference between NLFK and HeLa results. Membrane structures affect the *cytoplasm* diffusion coefficients in FRAP experiments, and they display strong variation. Evidently it is important to make a distinction in the interpretation of FRAP experiments between diffusion in the cytosol (nucleosol) and in the cytoplasm (nucleoplasm). Using a conventional modeling of the same FRAP experiments, much too low diffusion coefficients were found. The assumptions made in the conventional modeling were not realized in the experimental situation, and no difference was made either between cytosol and cytoplasm diffusion.

Without fine tuning the nucleus and nuclear envelope results we found by the new FRAP method that the 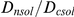 ratio was about a half in both cells. The nucleosol is thus a more molecularly crowded environment than the cytosol, in agreement with recent results [1]. Both 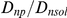 ratios were about two thirds. It appears that the solid phase affects protein diffusion less in the nucleoplasm than in the cytoplasm [8], [23].

For clarity we considered here pure diffusion, but the methods introduced can be extended so as to include interactions of proteins with cellular organelles.
